# Late gadolinium enhancement (LGE) on Cardiac MR is a powerful predictor of death and “hard” events in patients with hypertrophic cardiomyopathy (HCM)

**DOI:** 10.1186/1532-429X-14-S1-P161

**Published:** 2012-02-01

**Authors:** Sabha Bhatti, Abdul Hakeem, Karthikeyan Ananthasubramaniam

**Affiliations:** 1 Cardiology, Henry Ford Hospital, Detroit, MI, USA; 2 Cardiology, William Beaumont Hospital, Royal Oak, MI, USA

## Summary

Presence of scar as depicted by LGE on CMR is a strong predictor of death and hard clinical endpoints. LGE on CMR may hence be considered a significant variable in the risk prediction equation for patients with HCM.

## Background

Patients with hypertrophic cardiomyopathy (HCM) represent a heterogeneous group at risk of death and other cardiac events, necessitating further effective risk stratification. Late gadolinium enhancement (LGE) on cardiac MR (CMR) represents fibrosis and has emerged as a possible risk predictor of hard events in several small studies. We sought to evaluate the prognostic utility of LGE in patients with HCM by performing a meta analysis of available studies.

## Methods

PubMed, Cochrane Register of Controlled Trials, conference proceedings, and internet-based resources of clinical trials. Studies evaluating the prognostic utility of LGE on CMR with outcomes of interest including death, aborted sudden cardiac death (SCD) were included. We used the risk ratio (RR) with 95% confidence intervals (CIs) as the metric of choice for outcomes. Categorical variables were reported as percentages and continuous variables are presented as means +/- standard deviation. Weighted means were used for the pooled estimates of continuous variables. The pooled RR was calculated with the DerSimonian-Laird method for random effects. To assess heterogeneity across trials, we used the Cochran Q via a 2 test based on the pooled RR by Mantel-Haenszel, as well as the I2 statistic.

## Results

Four studies comprising of 1063 patients without history of septal ablation or myectomy with mean (weighted) age of 51+14 with 68% male subjects formed the study population. There were 634 (59%) patients in LGE+ group and 429 (41%) patients in LGE- group. Mean duration of follow up was 34.5 + 9 months (range 22-43 months). Presence of LGE+ was associated with increased risk of all cause mortality and surrogates of SCD At follow up, hard events occurred in 71 patients (12%) in the +LGE group and 10 events (2.3%) in the LGE- group translating into a relative risk of 3.61 (1.29, 10.05); p=0.01. There was little heterogeneity with regards to the end point (I2=41%)

## Conclusions

Presence of scar as depicted by LGE on CMR is a strong predictor of death and hard clinical endpoints. LGE on CMR may hence be considered a significant variable in the risk prediction equation for patients with HCM.

## Funding

None.

**Figure 1:**
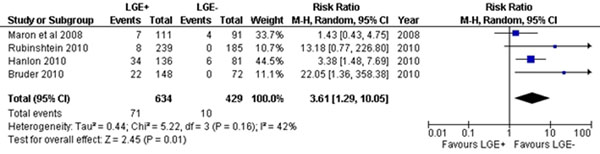
Forest Plot

